# Fluctuating biomarkers in primary sclerosing cholangitis: A longitudinal comparison of alkaline phosphatase, liver stiffness, and ELF

**DOI:** 10.1016/j.jhepr.2021.100328

**Published:** 2021-07-02

**Authors:** Guri Fossdal, Anders B. Mjelle, Kristine Wiencke, Ida Bjørk, Odd Helge Gilja, Trine Folseraas, Tom Hemming Karlsen, William Rosenberg, Lasse M. Giil, Mette Vesterhus

**Affiliations:** 1 Norwegian PSC Research Centre, Department of Transplantation Medicine, Division of Surgery, Inflammatory Diseases and Transplantation, Oslo University Hospital Rikshospitalet, Oslo, Norway; 2 Department of Clinical Science, University of Bergen, Bergen, Norway; 3 Department of Medicine, Haraldsplass Deaconess Hospital, Bergen, Norway; 4 Department of Clinical Medicine, University of Bergen, Bergen, Norway; 5 Section of Gastroenterology, Department of Transplantation Medicine, Oslo University Hospital, Oslo, Norway; 6 Research Institute of Internal Medicine, Oslo University Hospital Rikshospitalet, Oslo, Norway; 7 Department of Radiology, Oslo University Hospital Rikshospitalet, Oslo, Norway; 8 National Centre for Ultrasound in Gastroenterology, Department of Medicine, Haukeland University Hospital, Bergen, Norway; 9 Institute of Clinical Medicine, University of Oslo, Oslo, Norway; 10 UCL Institute for Liver and Digestive Health, University College London & Royal Free London NHS Foundation Trust, London, UK

**Keywords:** Primary sclerosing cholangitis, Alkaline phosphatase, Elastography, Liver stiffness, Enhanced liver fibrosis test, Biomarker, Risk stratification, ALP, alkaline phosphatase, ALT, alanine aminotransferase, AST, aspartate aminotransferase, CRP, C-reactive protein, ELF, enhanced liver fibrosis, FIB-4, Fibrosis-4 Index for Liver Fibrosis, GGT, gamma-glutamyl transferase, HA, hyaluronic acid, ICC, intraclass correlation, IgG4, immunoglobulin G4, INR, international normalised ratio, LSM, liver stiffness measurement, PIIINP, propeptide of type III procollagen, PSC, primary sclerosing cholangitis, pSWE, point shear wave elastography, ROI, region of interest, TE, transient elastography, TIMP-1, tissue inhibitor of metalloproteinases-1, UDCA, ursodeoxycholic acid, ULN, upper limit of normal

## Abstract

**Background & Aims:**

Primary sclerosing cholangitis (PSC) is a progressive liver disease characterised by fluctuating liver biochemistries and highly variable disease progression. The Enhanced Liver Fibrosis (ELF®) test and liver stiffness measurements (LSMs) reflect fibrosis and predict clinical outcomes in PSC; however, longitudinal assessments are missing. We aimed to characterise the systematic change in ELF and LSM over time in a prospective cohort of patients with PSC, along with their longitudinal relationship to alkaline phosphatase (ALP) and bilirubin.

**Methods:**

We included 113 non-transplant PSC patients (86 males [76.1%]; mean age 43.3 ± 15.7 years) with annual study visits between 2013 and 2019 at 2 Norwegian centres. ELF test, LSM, clinical data, liver biochemistries, and revised Mayo risk score were measured. We used linear mixed-effects models to estimate change over time, intraclass correlations (ICCs), and their relationship with ALP and bilirubin.

**Results:**

At baseline, the median (range) ELF test was 9.3 (7.5–12.9) and median LSM 1.26 m/s (0.66–3.04 m/s). ELF and LSM increased over time (0.09 point/year, 95% CI [0.03, 0.15], *p* = 0.005, *vs*. 0.12 point/year, 95% CI [0.03, 0.21], *p* = 0.009). Between-patient effects explained 78% of ELF variation (ICC 0.78) and 56% of LSM variation (ICC 0.56). ALP also increased and showed the highest ICC (0.86).

**Conclusions:**

ELF and LSM increased over a 5-year period. Longitudinal analyses demonstrated differences regarding within- and between-patient effects, suggesting that the ELF test may have superior reliability for risk stratification compared with LSM in PSC.

**Lay summary:**

Primary sclerosing cholangitis (PSC) is characterised by substantial disease variability between patients and fluctuating liver biochemistries. Hence, new biomarkers are needed to identify individuals with an increased risk of developing end-stage liver disease. We explore the change over time of 2 putative prognostic biomarkers in PSC, the serum Enhanced Liver Fibrosis (ELF®) test and LSMs by ultrasound, demonstrating differences that may reflect differing abilities to discriminate risk.

## Highlights

• ELF and LSM increased in patients with PSC, but only in patients with ALP >1.5× ULN.
• ELF may be more reliable for PSC risk stratification (low within-patient variation).
• A subgroup showed concomitant spontaneous reduction in ALP, ELF, and LSM.

## Graphical abstract

**Figure:**
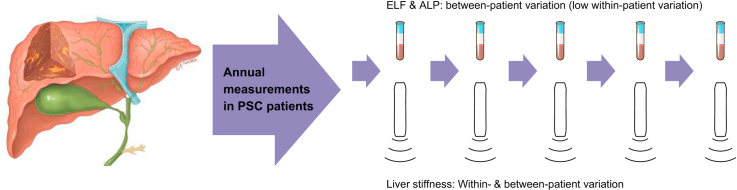

## Introduction

Primary sclerosing cholangitis (PSC) is characterised by multifocal strictures and dilatations of the biliary tree as a result of inflammation and biliary fibrosis, ultimately progressing to end-stage liver disease.[1], [2], [3] The natural course of PSC is highly variable, with median transplant-free survival ranging from 13 to 20 years.^2^^,^^4^^,^^5^ A major unmet need is the lack of established biomarkers to (a) gauge changes in disease activity that reflect the pathophysiological processes involved in PSC, (b) identify high-risk patients for risk stratification and prognostication, and (c) evaluate treatment effects before reaching clinical end points. Alkaline phosphatase (ALP) has been applied widely to predict clinical disease progression, to select patients for clinical trials, and as a surrogate outcome marker in treatment studies. Elevated ALP is a consistent marker of poor outcomes at the group level across several studies.[6], [7], [8], [9] However, longitudinal fluctuation in ALP limits its use at the individual level. Thus, there is a need to identify more accurate biomarkers with less fluctuation over time.

The Enhanced Liver Fibrosis (ELF®) test and liver stiffness measurements (LSMs) are emerging biomarkers for risk prediction and evaluation of treatment effects in clinical trials in PSC.^10^^,^^11^ They both reflect fibrosis severity but are based on different approaches. The ELF test is a serum-based biomarker panel measuring 3 direct markers of extracellular matrix remodelling and fibrosis.^12^^,^^13^ In contrast, LSM assesses the physical, viscoelastic properties of the liver using ultrasound-based elastography methods.^14^ Both the ELF test and LSM have been shown to predict transplant-free survival in PSC across independent studies.[15], [16], [17], [18], [19] However, studies assessing repeated measurements are limited and have not established whether ELF or LSM changes systematically over time in a similar fashion to each other or similar to ALP. Furthermore, it is not known whether ELF or LSM fluctuates together with ALP.

Therefore, we aimed to characterise the longitudinal change in ELF and LSM compared with ALP in a prospective cohort of patients with PSC. We also aimed to evaluate the relative contributions of intra- and interindividual variation for each of these variables using repeated measurements. Finally, we sought to establish the longitudinal associations between ELF, LSM, ALP, and bilirubin.

## Patients and methods

### Study design

We prospectively included 113 patients with PSC who did not undergo transplantation during 2013–2018 from 2 Norwegian centres: Haukeland University Hospital, Bergen, and Oslo University Hospital, Rikshospitalet, Oslo. The diagnosis of PSC was based on characteristic findings on magnetic resonance cholangiography or endoscopic retrograde cholangiopancreatography according to established diagnostic criteria.^20^ The first pathological radiologic finding defined the time of PSC diagnosis. Eight patients with PSC and features of autoimmune hepatitis were included. Patients with small-duct PSC were excluded. Inflammatory bowel disease was diagnosed based on endoscopy and histological findings according to accepted criteria.^21^ Clinical and demographic information, including laboratory data, was acquired from patient records and research databases. Liver biochemistry, ELF test, and elastography were sampled annually (±1 month from study visit) from the baseline visit. All patients provided informed written consent. The study was in accordance with the Declaration of Helsinki and approved by the Regional Committees for Medical and Health Research Ethics of Western and South-Eastern Norway (Reference 2012/2214/REK VEST and 2008/8670, respectively).

### Laboratory analyses

Biochemical analyses were performed following standard laboratory protocols, including haemoglobin, leucocytes, platelets, international normalised ratio (INR), aspartate aminotransferase (AST), alanine aminotransferase (ALT), ALP, gamma-glutamyl transferase (GGT), total bilirubin, albumin, creatinine, immunoglobulin G4 (IgG4), and C-reactive protein (CRP). The Mayo risk score and the Fibrosis-4 Index for Liver Fibrosis (FIB-4 score) were calculated using published algorithms.[22], [23], [24]

### ELF test

Frozen serum samples were collected from the 113 patients from 2 biobanks in Bergen and Oslo, following an identical protocol. The ELF test was analysed using the commercially available kit, Siemens ELF®Test, performed on an ADVIA Centaur XP analyser (Siemens Medical Solutions Inc., Tarrytown, NY, USA). The ELF test was calculated according to the published algorithm, including the levels of hyaluronic acid (HA), the propeptide of procollagen type III (PIIINP), and tissue inhibitor of matrix metalloproteinases-1 (TIMP-1), using the following formula: ELF test = 2.278 + 0.851 ln(C_HA_) + 0.751 ln(C_PIIINP_) + 0.394 ln(C_TIMP-1_).

### Elastography

Point shear wave elastography (pSWE) was performed using an ElastPQ® Philips iU22 (Philips Healthcare, Andover, MA, USA) scanner (software version 6.3.2.2, convex C5-1 probe) and ARFI® Siemens Acuson S3000 (Siemens Medical Solutions USA, Inc., Malvern, PA, USA), in the Bergen and Oslo cohorts, respectively. The examination was performed following international guidelines, including at least 3 h of fasting before examination.^14^ Following a B-mode ultrasound scan of the liver and spleen, LSM was measured using a right intercostal approach during relaxed mid-respiration breath-hold with patients in the supine position, with their right hand beneath the head.

A region of interest (ROI) representing a 0.5×1.5 cm sample volume was placed 2–6 cm below the liver capsule in an area where homogenous liver parenchyma could be visualised, avoiding large vessels and bile ducts. LSM was based on the median of 10 acquisitions and considered valid when the success rate was equal to or above 60%. LSM was measured in meters per second (m/s). The published cut-off value of 4.9 kPa (∼1.28 m/s) was used to stratify patients for subgroup analyses.^25^ Liver stiffness is expressed as shear wave speed (m/s) or converted into Young’s modulus using the equation kPa = 3[(ms^−1^)^2^].^14^ Each patient was followed by a single elastography platform.

### Statistics

Values of *p* <0.05 were considered statistically significant. Continuous variables were evaluated for approximate normality using Q–Q plots and presented as means and SDs or medians and IQRs as appropriate. Because of significant right skewness, logarithmic transformations were applied to liver biochemistries, ELF, and LSM. Transformation resulted in approximate normality as assessed by Q–Q plots, in line with the assumptions of parametric statistical models. The Mann–Whitney *U* test, Student’s *t* test, and the Chi-square test were applied as appropriate. Correlations at study baseline were tested using the Spearman rank correlation owing to the non-normality of variables and illustrated graphically as a correlation network.

We used a linear mixed model with an unstructured covariance structure for repeated measurement analyses with random intercept and random slope. Intraclass correlation coefficients (ICCs) were estimated from an empty-means linear mixed-effects model. We used a 2-step approach to characterise the associations between LSM, ELF, ALP, and bilirubin in a multilevel context. First, the random intercepts, slopes, and residuals from a multilevel model, either ALP or bilirubin, were estimated and scaled to z-scores. By standardising the variables to a mean of 0 and a standard deviation of 1, the biomarkers are on the same scale with comparable effect sizes. The resulting positive or negative z-score will represent the magnitude of increase or decrease, respectively, in the effect size for all variables. The z-scores were subsequently entered as predictors in a second multilevel model, where they represent between-person differences (random intercepts), between-person linear rate of change (random slopes), and fluctuations (the remaining residuals).^26^ For the relationship between LSM and ELF, we were able to fit a multilevel structural equation model with random intercepts only using both LSM and ELF as separate outcomes. We estimated the correlation between the intercepts and residuals, representing the between-person and within-person correlations. The model was adjusted for time in study. Missing values were assumed to be missing at random. Data were pooled for the 2 different elastography modalities as individual patient trajectories were followed longitudinally using a single platform; there were no significant differences between the 2 cohorts (*p* = 0.39).

*Post hoc* analyses were performed for defined subgroups. Subgroups for liver fibrosis stages F0–2 and F3–4 were defined using the published cut-off value of 4.9 kPa (∼1.28 m/s) for pSWE in PSC.^25^ For further subgroup analyses, the cohort was divided according to presumed high-risk profiles at baseline,[8], [9], [10]^,^^13^^,^^15^^,^^27^^,^^28^ that is, ALP ≥1.5× upper limit of normal (ULN); ELF level ≥9.8; and for discrimination between mild and advanced fibrosis corresponding to METAVIR score F0–2 *vs*. F3–4, LSM ≥1.28 m/s, as outlined in Table 1. The analyses were conducted using SPSS version 26 (SPSS Inc., 2016, Armonk, NY, USA) and STATA 16 (StataCorp. 2019, Stata Statistical Software: Release 16.1. College Station, TX: StataCorp LP) for all analyses. The correlation network was generated using the qgraph package in R (R Core Team [2017]. R: A language and environment for statistical computing. R Foundation for Statistical Computing, Vienna, Austria).

**Table 1:** **Baseline characteristics of the cohorts of patients with PSC**.

Demographics and clinical description	Total	Bergen	Oslo	Reference values	*p* value
Age at study start, x̄ (SD)	43.3 (15.7)	44.6 (16.0)	40.1 (14.6)		0.209
Age at diagnosis, x̄ (SD)	35.3 (14.8)	37.0 (15.1)	31.0 (13.0)		0.045
Males, n (%)	86 (76.1)	58 (71.6)	28 (87.5)		<0.001
PSC duration in years, M (IQR)	4.0 (11)	3.0 (13)	7.0 (9)		0.093
Mayo risk score, x̄ (SD)	-0.5 (0.9)	-0.5 (0.9)	-0.4 (1.0)		0.430
FIB-4 score, M (IQR)	1.1 (1.2)	1.2 (1.5)	0.9 (0.9)		0.808
Decompensated liver disease, n	2	1	1		0.251
Any inflammatory bowel disease, n (%)	85 (75.2)	62 (76.5)	23 (71.9)		0.627
Ulcerative colitis, n (%)	64 (56.6)	45 (55.6)	23 (71.9)		
Crohn’s disease, n (%)	12 (10.6)	10 (12.3)	2 (6.3)		
Indeterminate, n (%)	8 (7.1)	6 (7.4)	2 (6.3)		
UDCA treatment at any time, n (%)	39 (34.5)	25 (22.1)	14 (12.4)		<0.001
Patients with endoscopic intervention, n (%)	6 (5.3)	3 (3.7)	3 (9.3)		0.362
**Prognostic biomarkers**					
Participants above cut-off values					
ALP,∗ n (%)	52 (46)	36 (44.4)	16 (50)		0.362
ELF,† n (%)	37 (32.7)	22 (33.3)	10 (31.3)		0.428
LSM,‡ n (%)	50 (45)	37 (45.7)	13 (43.4)		0.098
Levels, M (IQR)					
ALP (U/L)	151.5 (197)	149.0 (196)	165.0 (206)	35–105	0.871
ALP by ULN, M (range)	1.4 (0.4, 8.0)	1.4 (0.4, 8.0)	1.5 (0.5, 6.1)		
ELF	9.3 (1.34)	9.3 (1.32)	9.4 (1.45)		0.905
LSM (m/s)	1.26 (0.52)	1.26 (0.48)	1.17 (1.21)		0.373
**Other blood tests, M (IQR)**					
ALT (U/L)	53.0 (81)	52.0 (66)	74.0 (127)	10–70 (m) 10–45 (f)	0.241
AST (U/L)	48.0 (49)	47.0 (48)	51.5 (75)	15–45 (m) 15–35 (f)	0.633
GGT (U/L)	228.0 (597)	149.0 (565)	238.5 (753)	10–80 (m <40 years)§ 10–45 (f <40 years)§	0.856
Bilirubin (μmol/L)	11.0 (10)	11.0 (9)	12.5 (16)	5–25¶	0.048
Thrombocytes (×10^9^)	245.0 (105)	240.0 (102)	240.0 (111)	145–390#	0.779
Albumin (g/L)	45.0 (5)	46.0 (5)∗∗	44.0 (5)††	see∗∗ and††	0.122

Reference values for laboratory parameters are equal for men and women and across study centres unless otherwise specified. P-values were calculated using Student's t-test, Mann-Whitney U test, or Chi-Square test as appropriate.

ALP, alkaline phosphatase; ALT, alanine aminotransferase; AST, aspartate aminotransferase; ELF, enhanced liver fibrosis; f, females; FIB-4, Fibrosis-4 Index for Liver Fibrosis; GGT, gamma-glutamyl transferase; LSM, liver stiffness measurement, M, median; m, males; PSC, primary sclerosing cholangitis; UDCA, ursodeoxycholic acid; ULN, upper limit of normal.

∗ ≥1.5× ULN.

† ≥9.8.

‡ ≥1.28 m/s.

§ GGT 15–115 U/L for m ≥40 years and 10–75 U/L for f ≥40 years.

¶ Bilirubin ≤21 μmol/L.

∗∗ Albumin 39–50 g/L for patients <40 years, 39–48 g/L for patients between 40 and 69 years, and 36–48 g/L for patients ≥70 years in the Bergen cohort.

†† Albumin 36–48 g/L for patients <40 years, 36–45 g/L for patients 40–69 and 34–45 g/L for patients ≥70 years in the Oslo cohort.

# Thrombocytes 145–348×10^9^ (m) and 165–387×10^9^ (f).

## Results

Patient characteristics are outlined in Table 1. We included 113 PSC patients (86 males; 76.1%). Their mean age at baseline was 43 years (SD 15.7), with a 4-year median duration of PSC and a median follow-up time of 4.5 years. Median time from study visit to LSM was 0 month (SD 1.33 and 2.33 for the Bergen and Oslo cohorts, respectively). Clinical events are listed in Table S1.

### Baseline ELF test, liver stiffness, and ALP values

**Fig. 1:**
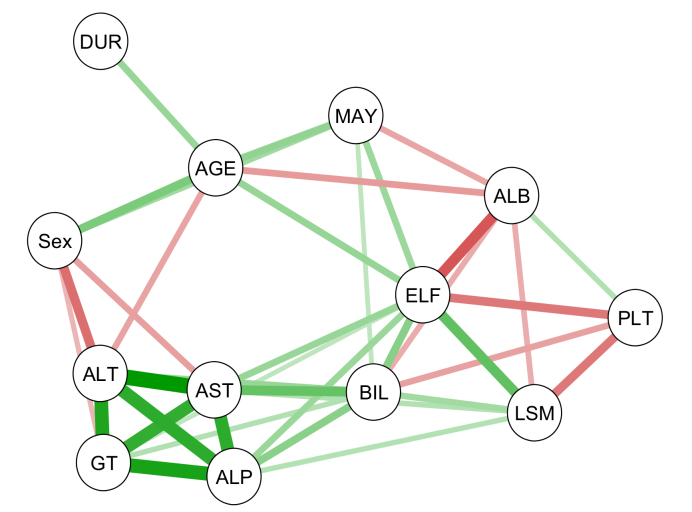
Correlation network for ELF, LSM, and relevant biochemistries. Correlations at study baseline were tested using the Spearman rank correlation. The strength of correlations is indicated by the widths of the connecting lines. Positive and negative correlations are represented by green and red colour, respectively. The diagram highlights liver enzymes ALT, AST, ALP, and GT as a group with high correlation. ELF and LSM were most strongly correlated with each other and showed correlations with liver enzymes and negative correlations with albumin and platelets. ALB, albumin; ALP, alkaline phosphatase; ALT, alanine aminotransferase; AST, aspartate aminotransferase; BIL, bilirubin; DUR, PSC duration; ELF, enhanced liver fibrosis; GT, gamma-glutamyl transferase; LSM, liver stiffness measurement; MAY, Mayo risk score; PLT, platelets; PSC, primary sclerosing cholangitis.

At baseline, the patients had median (IQR) ELF 9.3 (1.34), LSM 1.26 m/s (0.52), and ALP 151.5 U/L (197) (Table S2). There was no significant difference between males and females. There were 37 (33%), 50 (45%), and 52 (46%) high-risk patients defined by ELF test, LSM, and ALP, respectively. Correlation analysis showed a strong correlation of liver parameters, as illustrated by a network diagram (Fig. 1). The liver enzymes ALT, AST, GT, and ALP were strongly correlated; ELF and LSM showed moderate correlation with each other (rho 0.483, *p* <0.001), and both were correlated with ALP, other liver enzymes, bilirubin, and (negatively) albumin.

### Longitudinal change and ICCs

**Fig. 2:**
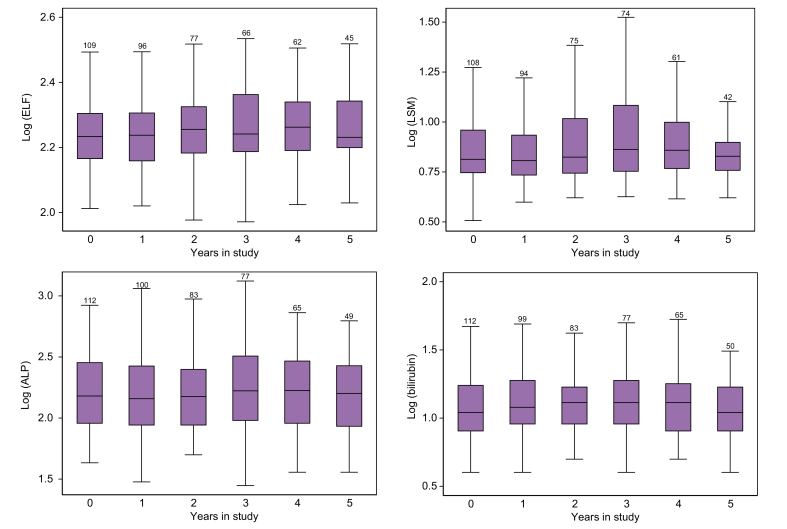
Development of ELF, LSM, ALP, and bilirubin over time in patients with PSC (n = 113). Boxplot; the lower and upper whiskers represent the first and third quartiles, respectively. Each box is represented by the number of measurements for each parameter per year in study. When applying a longitudinal mixed model analysis considering all available repeated measurements, there was a small but significant increase in ELF and LSM over time (*p* = 0.005 and 0.009, respectively). ALP, alkaline phosphatase; ELF, enhanced liver fibrosis; LSM, liver stiffness measurement; PSC, primary sclerosing cholangitis.

The development over time for the ELF test, LSM, ALP, and bilirubin is illustrated in Fig. 2. Using a linear mixed-effects model, we demonstrated a small but significant increase over 5 years for ELF (0.09 point/year, 95% CI [0.03, 0.15], *p* = 0.005) and LSM (0.12 point/year, 95% CI [0.03, 0.21], *p* = 0.009). Scaling of the outcome variables to z-scores demonstrated a slightly larger increase in LSM (0.07 SD per year, 95% CI [0.02, 0.13]) than in ELF (0.06 SD per year, 95% CI [0.03, 0.20]). By comparison, ALP increased by 0.04 SD per year (95% CI [0.01, 0.07], *p* = 0.011), and bilirubin increased by 0.07 SD per year (95% CI [0.02, 0.12], *p* = 0.007). The ICC was highest for ALP (0.86) and ELF (0.78), with lower ICCs for bilirubin (0.64) and LSM (0.56). The results are summarised in Table 2.

**Table 2:** **Liver stiffness measures and liver parameters over time**.

		Effect size	95% CI	*p* value
ELF	Fixed intercept∗	-0.11	[-0.29, -0.06]	0.196
	Fixed slope†	0.06	[0.02, 0.09]	0.005¶
	Crude ICC‡	0.78	[0.72, 0.83]	
	Adjusted ICC§	0.83	[0.77, 0.87]	
LSM	Fixed intercept∗	-0.11	[-0.27, 0.06]	0.199
	Fixed slope†	0.07	[0.02, 0.13]	0.009¶
	Crude ICC‡	0.56	[0.47, 0.65]	
	Adjusted ICC§	0.59	[0.48, 0.70]	
ALP	Fixed intercept∗	-0.03	[-0.21, 0.16]	0.775
	Fixed slope†	0.04	[0.01, 0.07]	0.011¶
	Crude ICC‡	0.86	[0.82, 0.89]	
	Adjusted ICC§	0.89	[0.85, 0.92]	
Bilirubin	Fixed intercept∗	-0.09	[-0.26, -0.09]	0.325
	Fixed slope†	0.07	[0.02, 0.12]	0.007¶
	Crude ICC‡	0.64	[0.55, 0.72]	
	Adjusted ICC§	0.71	[0.62, 0.78]	

ALP, alkaline phosphatase; ELF, enhanced liver fibrosis; ICC, interclass correlation; LSM, liver stiffness measurement.

∗ The fixed effect at baseline. All variables have been log-transformed and z-scored so that the mean represents the grand mean over 5 years. A negative fixed intercept indicates how much lower the variable is at baseline compared with the grand mean, in standard deviations.

† The fixed slope indicates change in the outcome in standard deviations per year.

‡ The ICC from an empty-means random intercept model.

§ The ICC from a random slope model adjusted for time-in-study.

¶ *p* value <0.05.

### Longitudinal change over time in high-risk subgroups

**Fig. 3:**
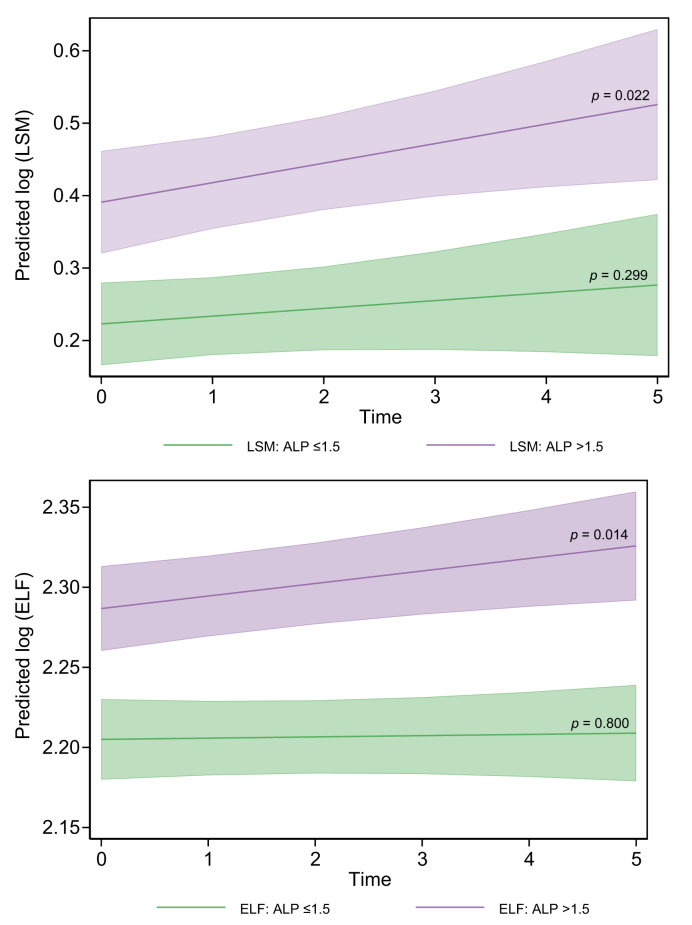
Linear mixed model analysis of the longitudinal development of ELF and LSM in high and low-risk groups defined by ALP. The high-risk subgroup (ALP ≥1.5× ULN at baseline) showed significantly higher baseline ELF (*p* = 0.001) compared with the low-risk group, with a similar trend for LSM (*p* = 0.06). Both ELF and LSM increased significantly over time in the high-ALP group (*p* = 0.014 and 0.022, respectively), whereas there was no significant increase for ELF or LSM in the low-ALP group. For ELF, there was a trend towards interaction between ALP-defined risk group and time which did not reach significance (*p* >0.05), whereas for LSM, there was no interaction between risk group and time (*p* >0.50). ALP, alkaline phosphatase; ELF, enhanced liver fibrosis; LSM, liver stiffness measurement.

*Post hoc* subgroup analyses of predefined high-risk groups, that is, ELF test ≥9.8, LSM ≥1.28 m/s, and ALP ≥1.5× ULN at baseline, demonstrated a significantly higher baseline ELF level among the high-ALP group compared with the low-ALP group (*p* = 0.001) and a similar trend for LSM (*p* = 0.06). Both ELF and LSM increased significantly over time in the high-ALP group (*p* = 0.014 and 0.022, respectively), whereas they showed no significant increase in the low-ALP group (Fig. 3). However, the interaction between time and the ALP subgroup did not reach significance. There were no significant differences in the change in ELF or LSM over time, according to the baseline risk groups defined by ELF or LSM (data not shown).

Ursodeoxycholic acid (UDCA) treatment was received by 35% of the patients at any time during the study with a median duration of 3.4 years (range 1–6 years) of treatment. Subgroup analysis indicated that ELF and ALP increased significantly over time in UDCA-naïve but not UDCA-treated patients (ELF: *p* = 0.009 *vs*. 0.803; ALP: *p* = 0.008 *vs*. *p* = 0.883), with a similar trend for LSM (*p* = 0.057 *vs*. 0.125); however, data were insufficient to adjust analyses for the biomarker × treatment interaction. Endoscopic interventions (n = 10 in 6 patients) during the study were not associated with consistent changes in ELF at subsequent visits.

### Longitudinal association between ELF and LSM

Using a multi-outcome multilevel structural equation model adjusted for time, we found that the correlation between the random intercepts of ELF and LSM was good (0.79, *p* <0.001), representing the between-person association between LSM and ELF. In contrast, the correlation coefficient of the residuals was weak (0.24, *p* = 0.007), representing the within-person association between LSM and ELF.

### Longitudinal association between ELF test or LSM and liver biochemistries and Mayo risk score

Over time, liver biochemistries and Mayo risk score were significantly associated with LSM and ELF outcomes (Table 3). ALP showed stronger association with ELF (standardised fixed effect [sFE] 0.47) than with LSM (sFE 0.28). Similarly, ELF showed a stronger association than did LSM with Mayo risk score (sFE 0.48 *vs*. 0.37) and the FIB-4 score (sFE 0.56 *vs*. 0.42). LSM was more associated with bilirubin (sFE 0.29) than was ELF (sFE 0.20), but ELF and LSM showed similar associations with albumin. The effect size sFE can be interpreted similarly in magnitude as correlation coefficients.

**Table 3:** **Associations of ELF and LSM with biochemical markers and clinical scores in a linear mixed-effects model**.

Predictor	Outcome	sFE∗	95% CI	*p* value
ALP	ELF	0.47	[0.37, 0.56]	<0.001
	LSM	0.28	[0.16, 0.39]	<0.001
Albumin†	ELF	-0.39	[-0.47, -0.32]	<0.001
	LSM	-0.35	[-0.44, -0.25]	<0.001
Bilirubin	ELF	0.20	[0.11, 0.29]	<0.001
	LSM	0.29	[0.18, 0.39]	<0.001
Mayo risk score†	ELF	0.48	[0.40, 0.56]	<0.001
	LSM	0.37	[0.26, 0.47]	<0.001
FIB-4	ELF	0.56	[0.46, 0.65]	<0.001
	LSM	0.42	[0.31, 0.53]	<0.001

Linear mixed-effects models as described under statistics.

ALP, alkaline phosphatase; ELF, enhanced liver fibrosis; FE, fixed effects; FIB-4, Fibrosis-4 Index for Liver Fibrosis; LSM, liver stiffness measurement; sFE, standardised fixed effects.

∗ sFE calculated as sFE = (FE×SD predictor variable)/SD dependent variable.

† Not log-transformed (all other log-transformed).

### Between- and within-person associations between ALP, bilirubin, LSM, and ELF

Variation in the individual means of ALP and bilirubin accounted for most of the association between ALP, bilirubin, and ELF (Table 4). By comparison, variation in the annual rate of change in ALP and bilirubin was not associated with ELF. However, we identified a smaller but significant association between fluctuations in ALP and ELF. For LSM, variation in individual means accounted for most of the association between ALP, bilirubin, and LSM, whereas there was no association with fluctuations in ALP or bilirubin. However, a higher annual rate of change in bilirubin was associated with higher LSM scores.

**Table 4:** **Decomposition of longitudinal associations of ELF and LSM with liver biochemistries in PSC**.

	Individual means (random intercepts)		Linear change (random slopes)		Fluctuation (residuals)	
	sFE (95% CI)	*p* value	sFE (95% CI)	*p* value	sFE (95% CI)	*p* value
**ELF as the outcome**						
ALP	0.37 (0.21, 0.52)	<0.001∗∗	0.03 (-0.14, 0.19)	0.768	0.15 (0.11, 0.18)	<0.001∗∗
Bilirubin	0.40 (0.26, 0.54)	<0.001∗∗	0.16 (-0.01, 0.31)	0.052	0.03 (-0.01, 0.08)	0.161
**LSM as the outcome**						
ALP	0.32 (0.18, 0.46)	<0.001∗∗	0.07 (-0.08, 0.21)	0.384	0.05 (-0.01, 0.11)	0.091
Bilirubin	0.42 (0.30, 0.54)	<0.001∗∗	0.23 (0.10, 0.35)	<0.001∗∗	0.03 (-0.04, 0.10)	0.407

A 2-step multilevel model where first the random intercepts, slopes, and residuals for the predictors ALP and bilirubin were estimated from separate models with time as the predictor. These now represent differences in individual means and individual linear rate of change, and the residuals represent fluctuating deviations from these. These were entered as predictors in a second multilevel model, with ELF or LSM as the outcome and time as the only covariate. ∗∗Statistically significant at *p* <0.001 level.

ALP, alkaline phosphatase; ELF, enhanced liver fibrosis; LSM, liver stiffness measurement; PSC, primary sclerosing cholangitis; sFE, standardised fixed effects.

### Spontaneous reductions in ELF, LSM, and ALP

The subpopulation with ALP ≥1.5× ULN accounted for all of the patients with ≥40% ALP reduction at each of the visits in our study. Out of the high-ALP group, a total of 13%, 13%, 10%, and 6% experienced ≥40% ALP reduction at visits 1, 2, 3, and 5 years from baseline, respectively.

In 40% of the total patient cohort, ELF levels decreased from baseline to 5 years, with a mean value of −0.67. A similar proportion of patients (44.7% and 42.2%) showed a reduction in ELF levels within the same range (mean change −0.51 and −0.54) at 1 and 2 years from baseline. Reduction in LSM was shown in 34% of the patients at 5 years (mean change −0.29 m/s); similar proportions of patients demonstrated LSM reduction at 1 and 2 years from baseline (42.7% and 36.7%, respectively; mean change of −0.33 to −0.38 m/s). Among the patients with 5-year follow-up time, all remained in the same category concerning low or high levels of ELF or LSM, whereas 16% of the patients moved between categories of low to high ALP as defined by ALP ≥1.5× ULN at baseline). At each follow-up visit (1–5 years from baseline), about 10% of patients featured a concomitant reduction in all of ELF, LSM, and ALP (Table S3), out of which only 25% received UDCA. Six patients received a total of 10 endoscopic treatments during the study period, of which only 2 procedures were followed by significant ALP reductions.

## Discussion

To our knowledge, this is the first study to provide an in-depth characterisation of the variation over time in ELF and LSM as well as ALP in a prospective cohort of patients with PSC, allowing differentiation of ‘background noise’ (random variation) from biological significant variation. ELF and LSM demonstrated a significant but minor increase over 5 years, in line with previous reports in patients with PSC and mild fibrosis.^9^^,^^17^^,^^27^ With the use of standardised z-scores in a linear mixed model, our results suggest that LSM increased more than ELF and ALP over time. We demonstrated a strong between-person association between LSM and ELF but a weak association for individual fluctuations over time. Overall, in this study, it was indicated that ELF and LSM may stratify similar patients to high-risk groups at baseline, whereas there may be different effects driving change in ELF and liver stiffness over time.

Using ICC analyses yielded by the mixed model, we demonstrated essential differences between ELF and LSM regarding between- and within-person effects influencing variation in these parameters. Whereas ELF showed high ICC, suggesting predominant between-person variation, between- and within-person variations contributed relatively equally for LSM. The relatively stable values within individual patients at repeated measurements for ELF support ELF as a reliable risk stratification marker and may imply that the ELF test is superior over LSM for risk stratification purposes when measured at a single time point. Biologically, this is plausible, as the ELF test reflects 3 direct markers of extracellular matrix remodelling, providing a biological link to disease severity, in contrast to LSM, which represents the sum of several factors affecting liver stiffness.

For a test to be useful for monitoring purposes, the ‘noise-to-signal ratio’ should be low; that is, any change should reflect a biological difference. Establishment of the magnitude of variation between and within patients is, therefore, a key factor for assessing the qualities of biomarkers. The ICC from the mixed model represents a measure of within- and between-variation in a test at a single time point and longitudinally. In general, a higher ICC value represents a lower degree of variation,^28^ reflecting a stronger ability to stratify risk between individuals at a single time point, whereas a lower ICC suggests higher sensitivity to biological variation over time, relevant for monitoring and assessment of treatment effect. However, interobserver variation and other factors may also contribute to lower ICC. Our findings are in line with quality assessments of ELF, which have shown good stability and a low coefficient of variation.^12^ The lower within-person variation for ELF compared with that for LSM may partly reflect the inherent differences between patented laboratory assays such as the ELF test compared with ultrasound-based LSM.

As a small note of caution, the ICC of ALP was higher than that of ELF, yet ALP is notoriously fluctuating over time in patients with PSC. This trait is a major challenge, limiting the use of ALP in individual prognostication and monitoring of disease activity. In the decomposed mixed model analysis, we identified concurrent fluctuations in ALP and ELF, which might suggest similar underlying mechanisms behind fluctuations in both parameters. Possibly, ELF may not overcome the problems of individual fluctuation typical for ALP. In favour of ELF towards LSM, we demonstrated stronger associations for ELF with ALP and other liver biochemistries, as well as the Mayo risk score and FIB-4 score.

For LSM, a lower ICC indicated that within-person variation explained a larger proportion of the variability compared with that for the ELF test, reflecting either improved sensitivity to detect biologically relevant changes or increased sampling variability. LSM has previously demonstrated good agreement towards histological stages of fibrosis and clinical outcome in PSC,[17], [18], [19], [29] and a strong predictive ability for clinical outcomes in independent studies.^17^^,^^18^ Moreover, the elastography modalities we used (pSWE and ARFI quantification) were reported to correlate well with histology^19^^,^[30], [31], [32] and demonstrated high accuracy in discriminating between lower and higher degrees of fibrosis[31], [32], [33] and excellent correlation to TE in patients with PSC.^25^ Because of lack of power for end-point analyses, we cannot decipher whether the larger relative contribution of within-patient effects on variability is a result of sampling variability or reflect biological variation over time. Inter and intraobserver variability is an acknowledged possible bias in all ultrasound-based methods.^25^^,^[34], [35], [36] Furthermore, the patchy disease distribution in PSC and variation in cholestasis may contribute to variations in LSM.^37^^,^^38^ Based on our results, we cannot rule out that the lower ICC for LSM results from increased measurement variability rather than reflecting a relevant change in fibrosis. The significant linear association between bilirubin levels and LSM over time but no association between their intermediate fluctuations indicates that limited segmental cholestasis in PSC does not severely affect LSM over time. This might suggest that ELF and LSM act as complementary biomarkers, indicative of slightly different aspects of the disease concerning fibrosis and cholestasis.

Interestingly, in a *post hoc* subgroup analysis, we found that patients with an ALP level ≥1.5× ULN at baseline demonstrated elevated baseline levels as well as a significant increase in ELF over time in the high-ALP compared with the low-ALP group. These findings support previous reports proposing this ALP level as an appropriate cut-off level for risk stratification.^6^^,^^7^^,^^39^

Clinical trials in patients with PSC are suffering from a lack of robust surrogate markers to reliably evaluate the effect of novel therapeutic agents. Reduction in ALP is commonly used as an outcome parameter in pharmacological studies; however, spontaneous reductions in ALP challenge the use of ALP as a surrogate marker in PSC.^7^^,^^8^^,^^39^^,^^40^ Although a reduction of ALP by 40% or more is a commonly applied primary outcome, this is questioned by reports of patients showing ALP reductions not supported by reductions in histological fibrosis.^9^ In the present study, we found that about 8% of the patients experienced spontaneous ALP reductions of at least 40% at 1, 2, and 3 years of study follow-up. These time points are commonly applied when designing clinical trials, underscoring the challenges of using ALP reduction as a surrogate endpoint. Furthermore, we demonstrated that between one-third and nearly one-half of the patients showed spontaneous reductions in ELF test and LSM, respectively, during the same time frame. Moreover, we identified a subgroup of about 10% of patients at each follow-up visit showing a concomitant reduction in ALP, ELF, and LSM, raising the question of whether the fibrosis level or disease stage may actually regress in PSC. These findings warrant further investigation before considering these biomarkers as surrogate endpoints in clinical trials.

UDCA treatment has been associated with ALP reduction in patients with PSC in clinical studies.^41^^,^^42^ We did not demonstrate ALP, ELF, or LSM reduction associated with UDCA; however, subgroup analysis showed significant increases in ELF and ALP over time in UDCA-naïve (65%) but not UDCA-treated (35%) patients. Moreover, UDCA users had higher levels of ELF, LSM, ALP, and bilirubin at baseline, suggesting a more advanced disease in this group. Unfortunately, our study was not powered to investigate biomarker × treatment interactions.

### Limitations of the study

The major limitation of this study is the limited number of long-term clinical outcomes such as deaths and liver transplantations, precluding end point analyses. Liver biopsies allowing direct assessment of the degree of liver fibrosis were also not available. However, in PSC, liver biopsies are poorly representative owing to the patchy disease distribution, and the procedure carries a risk of adverse outcomes. Current guidelines do not recommend liver biopsies; hence, this was considered unethical.

### Conclusion

The ELF test and LSM increased slightly but significantly over 5 years in a prospective panel of patients with PSC. Our longitudinal analyses demonstrated differences regarding within- and between-patient effects, suggesting that the ELF test may be more stable than LSM and is likely to perform better for risk stratification in PSC using single measurements. We advocate that the ELF test may hold practical utility for identification of PSC patients with a high risk of disease progression. ELF and LSM showed a significant increase over time only in patients with ALP≥1.5× ULN, supporting this as a relevant cut-off level for risk stratification. The significance of concomitant reductions in ELF, LSM, and ALP in a patient subgroup warrants further studies.

## Financial support

The work is part of the PhD program for GF funded by the 10.13039/501100004257Western Norway Regional Health Authority.

## Authors’ contributions

Guarantor of the article and supervised the project: MV. Conceived and designed the study: MV, LMG, WR, THK. Collected the biological samples and clinical data: MV, KW, TF. Performed the ultrasound scans and liver stiffness measurements: MV, ABM, IB. Contributed to the liver stiffness measurements: OHG. Contributed to the ELF test laboratory analyses: WR. Designed and performed the statistical analyses: GF, LMG. Contributed to the interpretation of the data.: GF, ABM, TF, THK, LMG, MV. Drafted the manuscript: GF, LMG, MV. Reviewed the manuscript for critical content and approved the final version of the manuscript: All authors.

## Data availability statement

Data is available upon request and an appropriate institutional collaboration agreement.

## Conflicts of interest

W. Rosenberg is one of the inventors and patent holders of the ELF test.

Please refer to the accompanying ICMJE disclosure forms for further details.
