# Supplementary material for: Fluctuating biomarkers in primary sclerosing cholangitis: A longitudinal comparison of alkaline phosphatase, liver stiffness, and ELF
Source: JHEP Rep. 2021 Jul 2;3(5):100328. doi: 10.1016/j.jhepr.2021.100328 (PMC8403583; doi:10.1016/j.jhepr.2021.100328)
Supplement: Multimedia component 1 [file mmc1.pdf]

Fluctuating biomarkers in primary sclerosing cholangitis: A longitudinal comparison of alkaline phosphatase, liver stiffness and ELF

Guri Fossdal, Anders Batman Mjelle, Kristine Wiencke, Ida Bjørk, Odd Helge Gilja, Trine Folseraas, Tom Hemming Karlsen, William Rosenberg, Lasse Melvær Giil, Mette Vesterhus

Table of contents

Fig. S1 2

Table S1 2

Table S2 3

Table S3 3

Fig. S1. Patients included.
The total number of patients included since initiation of the study.

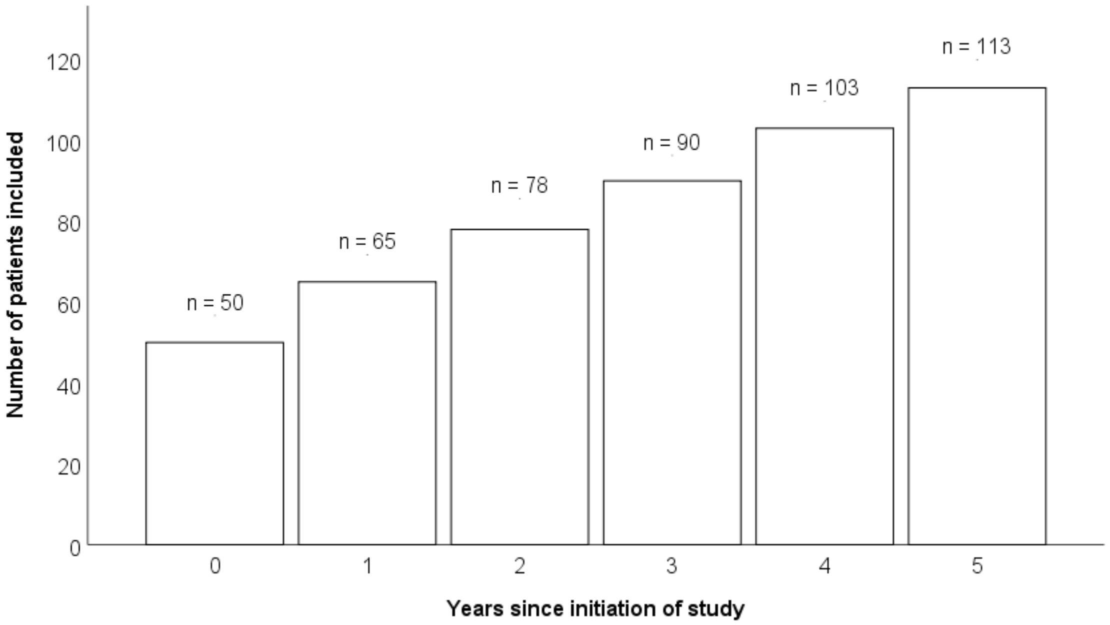

Table S1. Clinical events.
Number of patients in this PSC patient cohort undergoing clinical events in terms of liver transplantation or death since initiation of study.

**Indication for liver transplantation (n)**		**9**
	Biliary dysplasia	5
	Liver failure	2
	Cholangiocarcinoma	1
	Fatigue	1
**Deceased (n)**		**6**
	Liver failure	2
	Malignancies	2
	Post-transplant complications	1

Table S2. ELF, LSM, and laboratory values at annual visits.
All variables displayed as median (IQR).

	T0	T1	T2	T3	T4	T5
**N**	113	103	80	78	65	50
**ELF**	9.35 (1.35)	9.37 (1.43)	9.62 (1.37)	9.47 (1.77)	9.63 (1.58)	9.42 (1.40)
**LSM**	1.24 (0.49)	1.23 (0.46)	1.28 (0.63)	1.39 (0.91)	1.37 (0.58)	1.29 (0.36)
**ALP**	149 (198)	141 (178)	139 (141)	170 (244)	168 (230)	166 (170)
**ALT**	53 (81)	49 (83)	62 (106)	63.5 (85)	58.50 (86)	48 (69)
**AST**	45 (49)	44 (44)	50 (74)	61 (56)	55 (59)	55 (50)
**GT**	220 (611)	168 (432)	234 (532)	277 (472)	274 (403)	203 (411)
**Bilirubin**	11 (10)	12 (9)	13 (9)	14 (10)	13 (9)	11 (9)
**Albumin**	45 (5)	45 (4)	45 (5)	44 (5)	45 (5)	45 (5)
**Platelets**	237 (104)	229 (100)	227 (96)	231.5 (108)	208 (99)	

Abbreviations: ALP, alkaline phosphatase; ALT, Alanine aminotransferase; AST, Aspartate aminotransferase; ELF, Enhanced liver fibrosis; GT, Gamma-glutamyl transferase; LSM, Liver stiffness measurement;

Table S3 Spontaneous reduction in ALP, ELF, and LSM. Number of patients with spontaneous reduction in either ALP, ELF, LSM or all three parameters. Time represents year from baseline.

Time	1	2	3	4	5
ALP reduction, n (%)	46 (44.7)	39 (43.3)	35 (44.9)	28 (43.1)	28 (56)
ELF reduction, n (%)	46 (44.7)	38 (42.2)	23 (29.5)	24 (36.9)	20 (40)
LSM reduction, n (%)	44 (42.7)	33 (36.7)	24 (30.8)	26 (40)	17 (34)
Reduction in all three risk factors, n (%)	12 (11.7)	12 (13.3)	7 (9.0)	7 (10.8)	5 (10)

Abbreviations: ALP, alkaline phosphatase; ELF, enhanced liver fibrosis; LSM, liver stiffness measurement.
